# Evolution of NMDA receptor cytoplasmic interaction domains: implications for organisation of synaptic signalling complexes

**DOI:** 10.1186/1471-2202-9-6

**Published:** 2008-01-15

**Authors:** Tomás J Ryan, Richard D Emes, Seth GN Grant, Noboru H Komiyama

**Affiliations:** 1 Genes to Cognition Program, Wellcome Trust Sanger Institute, Cambridge, UK; 2 Institute for Science and Technology in Medicine, School of Medicine, Keele University, Staffordshire, UK

## Abstract

**Background:**

Glutamate gated postsynaptic receptors in the central nervous system (CNS) are essential for environmentally stimulated behaviours including learning and memory in both invertebrates and vertebrates. Though their genetics, biochemistry, physiology, and role in behaviour have been intensely studied *in vitro *and *in vivo*, their molecular evolution and structural aspects remain poorly understood. To understand how these receptors have evolved different physiological requirements we have investigated the molecular evolution of glutamate gated receptors and ion channels, in particular the *N*-methyl-*D*-aspartate (NMDA) receptor, which is essential for higher cognitive function. Studies of rodent NMDA receptors show that the C-terminal intracellular domain forms a signalling complex with enzymes and scaffold proteins, which is important for neuronal and behavioural plasticity

**Results:**

The vertebrate NMDA receptor was found to have subunits with C-terminal domains up to 500 amino acids longer than invertebrates. This extension was specific to the NR2 subunit and occurred before the duplication and subsequent divergence of NR2 in the vertebrate lineage. The shorter invertebrate C-terminus lacked vertebrate protein interaction motifs involved with forming a signaling complex although the terminal PDZ interaction domain was conserved. The vertebrate NR2 C-terminal domain was predicted to be intrinsically disordered but with a conserved secondary structure.

**Conclusion:**

We highlight an evolutionary adaptation specific to vertebrate NMDA receptor NR2 subunits. Using *in silico *methods we find that evolution has shaped the NMDA receptor C-terminus into an unstructured but modular intracellular domain that parallels the expansion in complexity of an NMDA receptor signalling complex in the vertebrate lineage. We propose the NR2 C-terminus has evolved to be a natively unstructured yet flexible hub organising postsynaptic signalling. The evolution of the NR2 C-terminus and its associated signalling complex may contribute to species differences in behaviour and in particular cognitive function.

## Background

One of the most striking evolutionary adaptations of mammals is their capacity for learning and memory [1]. Such higher cognitive abilities are generally attributed to increased brain size and elaborations of cortical structure [2]. However the contribution that evolution of molecular complexity of the synapse has made to cognitive properties of higher organisms has yet to be fully assessed [3].

The *N*-methyl-*D*-aspartate (NMDA) receptor is a class of glutamate gated transmembrane ion channel that functions at the postsynaptic membrane of excitatory synapses [4]. When opened, Ca^2+ ^influx through the NMDA receptor into the postsynaptic cell activates second messengers responsible for long lasting synaptic plasticity. Genetic and pharmacological studies have shown that NMDA receptors are necessary for normal spatial learning and its physiological correlate of long term potentiation (LTP) [4,5]. In addition, NMDA receptors have been shown to be essential for activity dependant brain development, in particular in the somatosensory and visual cortices in adults [6].

**Figure 1:**
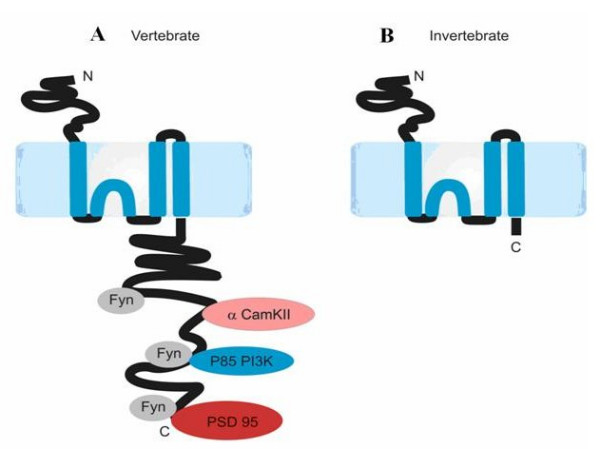
**Model of NR2 Subunit**. 1.A: Schematic of vertebrate NR2A or NR2B subunits. Note the long intracellular C-terminal domain and relative positioning of particular interacting proteins. PSD-95 is a scaffolding protein, while CaMKII and P13K are kinases that phosphorylate the NMDA receptor. 1.B: Schematic of invertebrate NR2. Note the significantly shorter intracellular C-terminus.

Each NMDA receptor is a tetramer formed by two heterodimers of an obligatory NR1 subunit and one of four possible NR2 subunits (NR2A, NR2B, NR2C, NR2D) [4]. Each subunit consists of an extracellular N-terminal domain with three transmembrane regions (M1, M3, and M4) and one re-entrant loop (M2) that form the channel pore, and an intracellular C-terminal domain (Fig. 1A). The N-terminal domains contain the ligand binding site, while the C-terminal domain interacts with intracellular signalling messengers including MAGUK (membrane associated guanylate kinase binding) proteins via a PDZ binding domain at its C-terminus. Binary protein-protein interaction studies have identified a range of scaffold proteins and enzymes that bind to the C-terminal domain [7], and proteomic studies have found that these proteins are assembled into 1–2 MDa size macromolecular signalling complexes known as NRC (NMDA Receptor Complexes) or MASC (MAGUK Associated Signalling Complexes) (Figure 1) [8,9].

Vertebrates contain four NR2 subunits with sequence similarity that suggests they are paralogues. In contrast a single NR2 orthologue is detectable in the invertebrate lineage, supporting the view that the four NR2 subunits seen in vertebrates are paralogues and arose from gene duplication events. Each NR2 paralogue has distinct spatial and temporal expression patterns [10,11]. NR2A and NR2B are expressed throughout the forebrain, including the hippocampus. NR2C is restricted to the cerebellum and NR2D is found predominantly in the midbrain. Developmentally, NR2B is expressed both embryonically and postnatally. NR2D is primarily embryonic while NR2A and NR2C are exclusively postnatal. Phenotypes observed after *in vivo *mutation of each NR2 subunit in mice reflect the brain regions they are expressed in [10-12]. Knockout (KO) of NR2A results in a deficiency in both spatial learning and LTP, confirming its essential function in the hippocampus [13]. NR2B shows the most severe phenotype of neonatal death at P0 due to lack of suckling response as well as abnormal brainstem trigeminal nucleus formation, showing its importance during development of the forebrain [14]. NR2C KO causes a decrease in NMDA receptor mediated currents in the granular cells of the cerebellum, and NR2A/NR2C double KOs result in impaired motor coordination [15,16]. Thus it is clear that since NR2 gene duplication each paralogue acquired discrete physiological functions and discrete developmental properties, both spatially and temporally.

The object of interest here is the intracellular 'tail' of the NR2 subunits, which lies in the C-terminal domain. Our impetus arises from its functional significance, originally demonstrated in knockin mouse models where the C-termini of NR2A, NR2B, and NR2C were deleted [17]. The C-terminal deletions resulted in behavioural and electrophysiological phenotypes identical to the null mutants, but without interfering with NMDA receptor channel formation and gating. Thus the intracellular signalling capacity of NR2 is indispensable for activity dependant NMDA receptor function. Interestingly, recent research suggests that differences in the intracellular signalling capacities of NR2 subunits may account for their contrasting physiological roles in induction of synaptic plasticity (LTP) and in neurotoxicity [18,19]. These studies have been strongly disputed due to lack of subunit specificity of NMDA receptor pharmacological antagonists [20,21]. Nevertheless, NR2 subunit specific interaction and phosphorylation sites have been identified and imply that NR2 subunit specific properties may me due to C-terminal variation [22]. The profound phenotypic effect of the C-terminal deletions may well be due to protein-protein interactions between the C-terminal domain and postsynaptic signalling molecules. We examined the intracellular domain of the NR2 sub-units using *in silico *methods and infer striking evolutionary adaptations with unique structural properties.

## Results

### Molecular evolution of NR2

**Figure 2:**
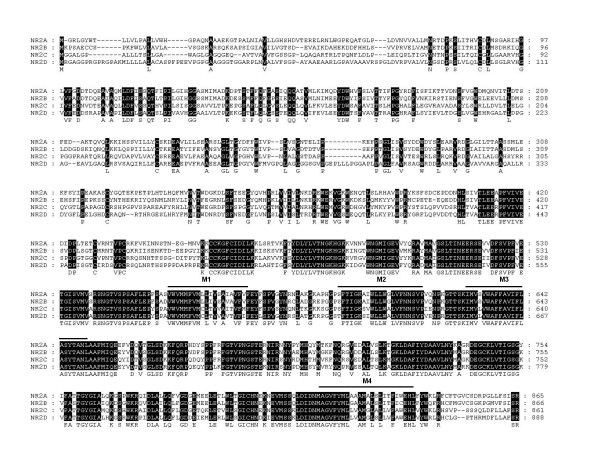
**NR2 Subunit Extracellular and Transmembrane Domain Alignment (Mouse)**. 100% consensus between sequences shown by black shading. Transmembrane domains (M1, M3, and M4) and re-entry loop (M2) are overlined.

We assessed the similarity of the mouse NR2 subunit amino acid sequences by multiple sequence alignment. It was clear that all four NR2 paralogues show a very high degree of similarity in their extracellular and transmembrane domains, most significantly NR2A and NR2B with 69% identity (Fig. 2). Such a high degree of conservation implies that they are indeed of common origin, and share the same transmembrane topology. Therefore the NR2 paralogues have remained under purifying selection since duplication of the ancestral NR2 and are likely to retain common function in their extracellular and transmembrane domains.

**Figure 3:**
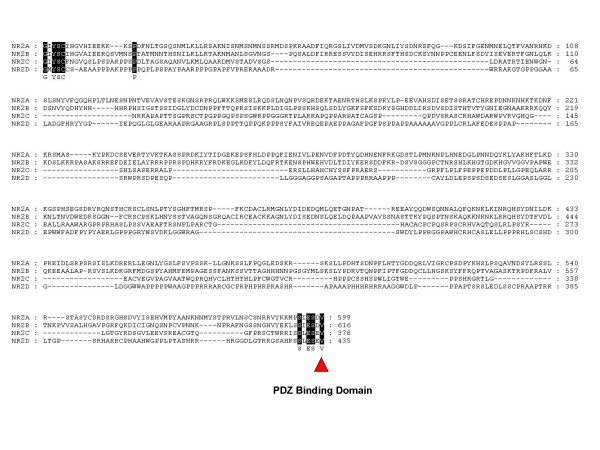
**NR2 Subunit C-terminal (intracellular) Domain Alignment (Mouse)**. 100% consensus between sequences shown by black shading. PDZ binding domain indicated by red arrow.

**Figure 4:**
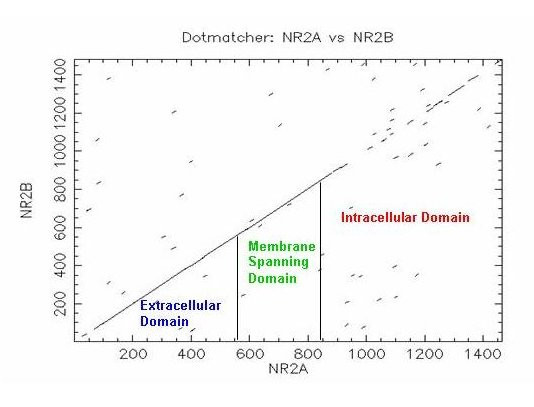
**Dotplot of NR2A and NR2B (mouse)**. N-terminal extracellular domain and membrane spanning domain show high similarity. The C-terminal tail region has very low sequence similarity. C-terminal domain begins at AA position 866.

In contrast to these domains, the C-terminal intracellular domains of the NR2 paralogues have diverged significantly, with only 9 residues conserved between the four paralogues, primarily at the extremities of the C-terminal domain surrounding an internal cassette (Fig. 3). The most similar NR2 paralogues in pairwise comparisons are NR2A and NR2B, which show 29% identity in their C-terminal domains. Although this is a significantly high similarity, it is relatively low in comparison to the N-terminal regions. When compared on a Dotplot it is clear that their C-terminal domains differ profoundly relative to their N-terminal domains (Fig. 4). This indicates that since duplication in the vertebrate lineage, the NR2 subunits have retained common ancestral structure in their extracellular and tramsmembrane domains but diversified in their intracellular domains, suggesting specific functional adaptations in intracellular signalling properties for each paralogue.

**Figure 5:**
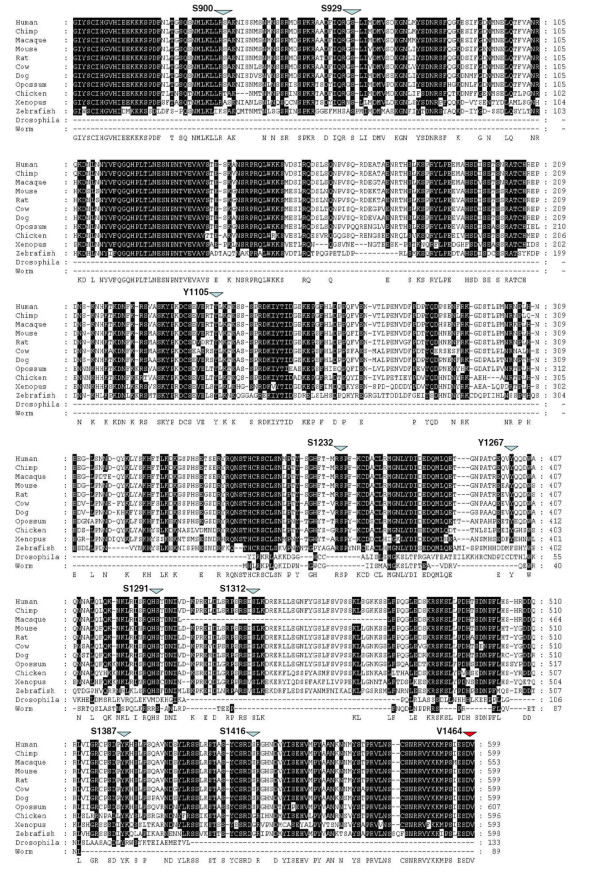
**Alignment of NR2A C-terminal Domain Across Species**. 80% consensus between sequences shown by black shading. Phosphorylation sites indicated by blue arrows. Vertebrate PDZ binding domain indicated by red arrow. Invertebrate PDZ binding domain (T-V/N-L) is present at the C-terminus of the Fly and Worm sequences.

In order to investigate the evolution of the ancestral C-terminal domain of NR2, alignments were performed with sequences of various species representative of metazoan clades including the NR2 protein sequences of invertebrates (*C. elegans*, *D. melanogaster*,); and the NR2A protein sequences of non-mammalian chordates (*D. rerio*, *G. gallus*, *X. Tropicalias*); and mammals (*M. domestica*, *C. familiaris, B. Taurus, R. norvegicus*, *M. musculus*, *M. mulata, P. troglydytes *and *H. sapiens*) (Fig. 5). From this alignment it was strikingly apparent that vertebrate C-terminal domains were substantially longer than those of invertebrates. It was also clear that, although there is much diversity between NR2 paralogues within a species within the central portion of the NR2 C-terminus, there were many regions of conservation between species for each NR2 subunit (Fig. 5). Strikingly, the few amino acids that are conserved between the four paralogues lie in short 'islands' at the very beginning of the C-terminus (at G-I/M-YSC motif at the beginning of the terminal exon) and at the very end where the PDZ binding domain lies. This suggests that these regions act as necessary 'sockets', one for the transmembrane domain of the NMDA receptor upstream, and the other for interacting with PDZ containing proteins downstream [23].

**Figure 6:**
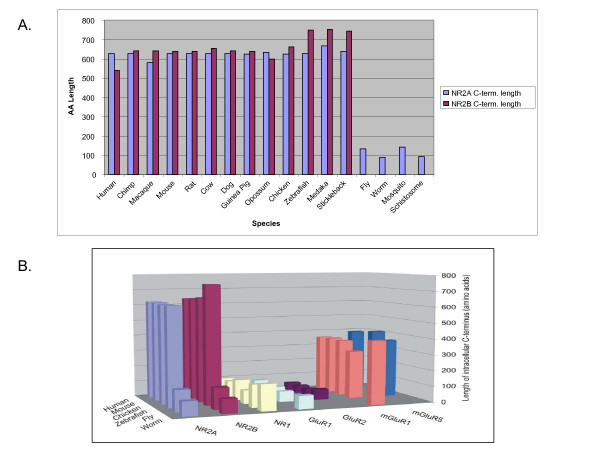
**Comparison C-termini Length Across Species**. 6.A: Comparison of NR2A and NR2B C-termini Length Across Representative Species. Lengths in amino acid number were calculated from the position of the most distal transmembrane domain, as predicted by TMHMM. The longer NR2 can be seen to be Vertebrate Specific. 6.B: Species Comparison of Glutamate Receptor C-terminal Domain Length. Lengths in amino acid number were calculated from the position of the most distal transmembrane domain, as predicted by TMHMM. The vertebrate/invertebrate contrast in length of the C-terminal region is specific to NR2.

When the lengths of the NR2 C-terminal regions (defined by the amino acid sequence starting downstream of the last predicted transmembrane amino acid) were compared across the above species, as well as *S. mansoni*, *A. aegypti*, *G. aculeatus*, *C. porcellus*, and *O. latipes*, it was noted that the NR2 of all sequenced invertebrate species are substantially shorter by up to >400 amino acids than vertebrate NR2A/B and >200 amino acids for NR2C/D, highlighting a stark contrast in the intracellular domain of NMDA receptors between vertebrates and invertebrates (Fig. 1B, Fig. 6A). The invertebrate NR2 C-terminal domain ranges from 89 residues in *C. elegans *NR2 to 133 residues in *D. melanogaster *NR2, whereas the vertebrates NR2A/B 'tails' range from 581 in *M. mulatta *NR2A to 754 residues in *Oryzias latipes *(Medaka) NR2B. Within the vertebrate clade the length of NR2 C-termini does not significantly vary with the exception of NR2B in Teleosts, which is predicted from genomic sequence to carry twenty insertions. Teleosts have two copies of each NR subunit, most likely as a consequence of the whole genome duplication that occurred in that lineage [24], and which may have allowed for relaxation of constraint on the second NR2B copy.

Since the NMDA receptor is comprised of NR1 and NR2 subunits, we next asked if NR1 also showed marked differences in length between invertebrates and vertebrates. This was not the case (Fig. 6B) and we therefore inspected a range of other glutamate receptor subunits including ionotropic AMPA receptors (e.g. GluR1) and metabotropic receptors (e.g. mGluR1). In mammals these receptor subunits are known to link via their C-termini to adaptor proteins forming complexes bringing together these different classes of glutamate receptors. Our results show that an elongated vertebrate C-terminal domain is unique to the NR2 glutamate receptor subunit (Fig. 6B).

To search for the origin of the vertebrate NR2 C-terminus, the nucleotide sequence of each mouse NR2 subunit C-terminal domain was run in BLASTn and tBLASTn searches against all available genomic sequences, ESTs, and non-redundant databases. In addition the amino acid sequence of the four mouse NR2 C-terminal domains were used to search the Pfam database for any domain that might elucidate its origin or function [25]. No hits of significance were found in either case. Exhaustive searches of mouse NR2 C-termini with invertebrate genomes could not identify any homologous gene or similar sequence.

**Figure 7:**
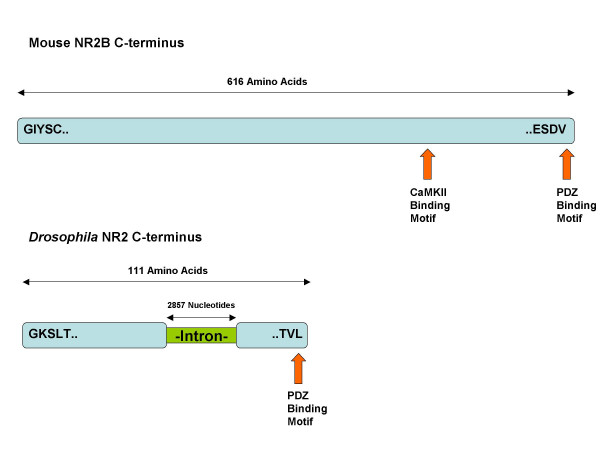
**Comparison of the Amino Acid Translation of the C-terminal exon of Mouse NR2B with the corresponding region from *Drosophila***. CaMKII phosphorylation site on mouse NR2B indicated by blue arrow. PDZ binding motifs indicated by red arrows.

Since BLAST searches of invertebrate genomes using the mouse C-terminal NR2 protein sequence failed to find a similar region, we inspected the NR2 invertebrate genomic locus more closely. We had observed that the NR2 C-terminus was noted to have conserved exonic structure throughout vertebrates, with the vast majority of the NR2 C-termini being encoded for in the terminal exon. This conservation of genomic structure was not seen in invertebrates, where the genomic sequence of the NR2 C-terminus is interrupted by introns in *D. melanogaster*, *C. elegans*, and *S. mansoni*. In the case of *D. melanogaster *there are two introns seperating the NR2 C-terminal domain nucleotide sequence of 114 and 2857 nucleotides in length, respectively (Fig. 7). When the nucleotide sequence of mouse NR2B terminal exon was aligned with the 2857 nucleotide intron of *D. melanogaster *NR2 C-termini, 41.6% identity was found by conducting a local Smith-Waterman alignment (additional file 1). Although this is not a strong result at the nucleotide level it may imply that the sequences have a common origin. A similar result (42.1%) was obtained using the corresponding NR2 C-terminal intronic sequence of *S. mansoni *(data not shown).

### Structural aspects of NR2 C-terminal domain evolution

**Figure 8:**
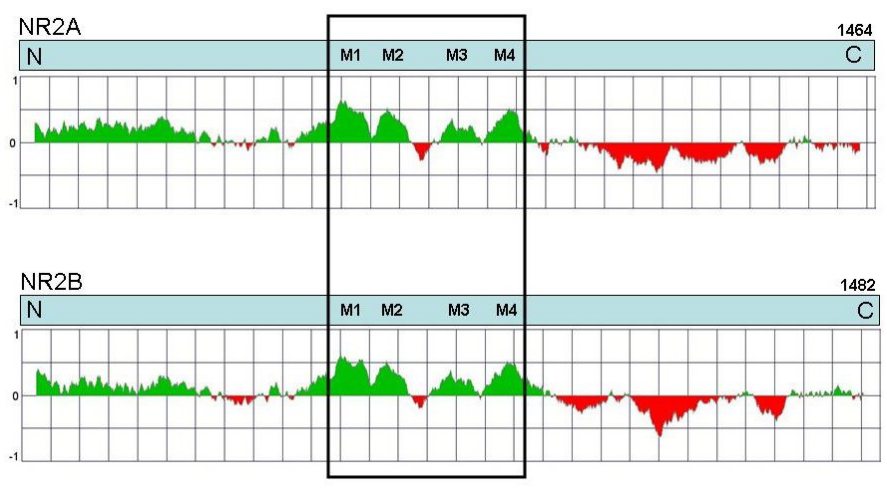
**FoldIndex Comparison of NR2 C-terminal Domain Folding Propensity**. Plot of AA sequence of N-terminus (N) to C-terminus (C) of mouse NR2A and NR2B against probability of folding. Green areas are predicted to be intrinsically folded, red areas are predicted to be intrinsically unfolded. Transmembrane regions (M1, M3, and M4) and re-entry loop (M2) are boxed.

Since the C-terminal domain of NR2 functions by attaching to, and organising intracellular adaptor and signalling molecules it is possible that the diversity between NR2 paralogues is manifest at the level of protein structure. The amino acid composition of each subunit was calculated and it was noted that, when compared to all other constituent amino acids, NR2A and NR2B were enriched for serine, 13.2% and 12.2%, respectively, while NR2C and NR2D were substantially enriched for proline at 14.9% and 21.8%, respectively. It has been reported that an amino acid composition of >6.91% serine or >12.07% proline is indicative of intrinsically disordered proteins [26]. We therefore used a graphic web server FoldIndex to examine the four mouse NR2 paralogues to further predict protein folding propensity (Fig. 8, additional file 2) [27]. A large portion of N-terminal extracellular part shows a relatively high probability for folding, four peaks of very high probability correspond to the four transmembrane segments of the NR2s. In contrast to the extracellular and transmembrane domains the entire C-terminal cytoplasmic tail was predicted to be intrinsically unfolded for all four NR2s. This indicates that the intracellular tail of NMDA receptors may under some conditions remain unfolded.

**Figure 9:**
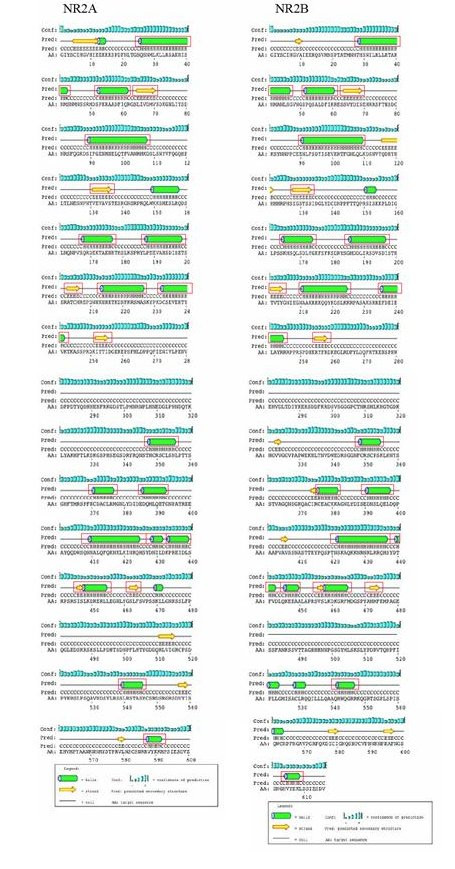
**PSIPRED Secondary Structure Predictions of Mouse NR2A & NR2B C-terminal Domains**. Green barrels represent alpha helices while yellow arrows signify beta sheets. Prediction confidence represented by blue bars. Structural motifs occurring in equivalent locations in NR2A and NR2B are boxed in red.

Since the NR2 C-terminus is known to bind directly to various interacting proteins, it might be expected that it could adopt a secondary or tertiary structure, and it is of interest to compare the structure of the different NR2 subunits. In the absence of 3D structure we applied the PSIPRED Protein Structure Prediction Server to compare the computationally predicted secondary structure of NR2A and NR2B amino acid sequences (Fig. 9) [28]. Surprisingly, though NR2A and NR2B C-termini show low primary sequence identity relative to the rest of the protein (29%, Fig. 4), they exhibit strikingly similar secondary structure, where the relative positioning of alpha helices and beta sheets is conserved (Fig. 9). PSIPRED was also applied to NR2C and NR2D but their secondary structure predictions showed similarity neither to each other nor NR2A or NR2B (additional file 3).

## Discussion

### Evolutionary features of the NMDA receptor intracellular C-terminal domain

We used bioinformatic tools to examine the evolutionary origins of the NR2 intracellular domain. Though it has previously been observed that the four NR2 paralogues found in vertebrates differ in size due to variation in the length of their C-terminal domains, the overall level of conservation between them has not been addressed [4]. We found that there is a profound level of diversity between the NR2 paralogues, specifically in their C-terminal intracellular domains. In contrast their N-terminal extracellular regions are highly conserved. Generation of a phylogenetic tree using the conserved central part of the NMDA receptor produces a topology grouping with the NR2A/NR2B subunits in one clade, and the NR2C/NR2D paralogues in another (additional file 4). The C-termini alignment reinforces this grouping since NR2A and NR2B have clearly longer C-termini while NR2C and NR2D are shorter. Additionally, NR2A and NR2B C-termini show a significant degree of conservation (29%) and are serine rich, while NR2C and NR2D (19% identical) are highly enriched for proline. The two sub groups imply that two separate duplications of NR2 occurred at the base of the vertebrate lineage, with the first giving rise to proto-NR2A/B and proto-NR2C/D paralogues. These have subsequently duplicated and diverged, giving rise to the four NR2 paralogues found in extant vertebrates. Both rounds of duplication occurred following divergence from the urochordates, and are likely a consequence of the whole genome duplication that took place in early chordate evolution [29].

Based on multiple species alignments of NR2 orthologues we show that vertebrate NR2s are consistently longer than invertebrate NR2s, and that this is due to difference in C-terminal domain length. We next asked if this vertebrate specific intracellular extension was a feature of the NR1 subunits or other glutamate receptors including ionotropic AMPA (Glur1, Glur2) and metabotropic receptors (mGluR1, mGluR5). The C-termini of all the other mentioned glutamate receptors do not exhibit comparable extensions between invertebrates and vertebrates. Thus the vertebrate/invertebrate contrast is specific to NR2. All of these glutamate receptor subtypes are involved with synaptic plasticity and are linked via C-terminal domains to intracellular adaptor proteins.

The ~5 fold difference in NR2 length between vertebrates and invertebrates may in principle result from either a vertebrate specific elongation or an invertebrate specific truncation. Since only two clades are available for comparison here (invertebrates and vertebrates) it is not possible to distinguish between the ancestral and the derived state. The most parsimonious explanation for the NR2 length difference is a loss of function in the common ancestor of invertebrates, since a single random mutation introducing a stop codon into the NR2 of a common ancestor of invertebrates would be sufficient to truncate the NR2 C-terminus. However this is an unlikely mechanism for shortening of the C-terminus since both the invertebrate and each of the vertebrate NR2 subunits have a type I PDZ binding domain at their carboxyl termini [30,31]. We noted that the NR2 C-terminus has a conserved exonic structure throughout vertebrates, with the vast majority of the NR2 C-termini being encoded for in the terminal exon, which is in contrast to invertebrates where it is coded by multiple exons (and the intron/exon junctions do not show conservation within invertebrates). Interestingly, the principal intron of the *D. melanogaster *NR2 C-terminus bears some similarity (42%) to the terminal exon of mouse NR2B suggesting that NR2 may have been internally truncated due to intron gain in a common ancestor of invertebrates. Alternatively the similarity seen in the *D. melanogaster *intron may imply a vertebrate specific elongation perhaps due to the intron becoming coding, but this may be less likely due to the presence of in-frame stop codons in an intron. The ancestral state of NR2 may be revealed by further genome sequencing of more diverse and complex invertebrates. If Lophotrochozoans were found with the elongated NR2 version, then a C-terminal truncation in the Ecdysozoan clade would be the most parsimonious explanation [32], and if the shorter form was unambiguously identified in a Urochordate genome then a vertebrate specific elongation would be supported [33].

### Evolution of signalling complexes and behavior

In comparison with invertebrates, the more diverse family of vertebrate NR2 subunits and their longer NR2 C-termini could have significant implications on the intracellular signalling capacity of the NMDA receptor channel. At the anatomical level, the duplication of the elongated NR2 in the vertebrate common ancestor has allowed for each paralogue to diverge and establish distinct but overlapping anatomical and developmental roles in the mammalian brain by subfunctionalization of gene duplicates [10,34]. At the level of signal transduction complexes, we propose that the longer vertebrate C-terminus allowed a greater NMDA receptor dependant signalling complexity to evolve in vertebrates.

It is established that the mammalian NMDA receptor is in close contact with plethora of proteins that form the NRC/MASC (Fig. 1) [8,35]. It is reasonable to predict that the invertebrate NR2 C-terminus would bind to a smaller set of postsynaptic proteins: invertebrate NR2 is far shorter and has only one identifiable interaction motif (the PDZ binding domain). If an elongated NR2 intracellular domain did exist in a common ancestor of invertebrates, then we predict that those proteins within the NRC/MASC that bind mammalian NR2 C-terminus would be found in invertebrates. Indeed, CaMKII [36], alpha-actinin 2 [37], PLC-gamma [38], Rack1 [39], Grit [40], Clathrin Adapter Protein 2 [41], and the MAGUKs [30] are all molecules that directly interact with the NMDA receptor in vertebrates and have an orthologue in invertebrates. Moreover, of the 186 mammalian NRC/MASC proteins there are 91 identifiable orthologues in the *Drosophila melanogaster *genome (data not shown). These observations suggest that many vertebrate NMDA receptor interactions with cytoplasmic signalling proteins are not important to the physiology and behaviour of invertebrates.

The expansion of the NR2 gene family has been utilised in the mammalian brain to produce distinct functional roles. Studies of synaptic plasticity at the CA3-CA1 synapses of the hippocampus, using mice lacking these subunits or with subunit specific pharmacological antagonists, reveal differences in synaptic plasticity [12]. As mentioned above, the subunits are expressed at different times and locations during development of the nervous system implying that their specific signalling functions have been exploited to serve roles in organisation of the anatomically complex mammalian brain. The mammalian MAGUK family of proteins, which bind to the PDZ binding domain at the C-terminus of NR2, shows a similar evolutionary pattern of duplication followed by subfunctionalisation, with diverse expression and function between the four paralogues (PSD-95, PSD-93, SAP-102, and SAP97). The duplication of MAGUKs in vertebrates complements the NR2 duplication, resulting in potentially sixteen binary configurations of NR2-MAGUK interactions, substantially increasing the level of NMDA receptor interaction complexity in vertebrates. The specificity of these interactions has been shown to be crucial to cognitive function in mammals, as SAP-102 deficient mice show mild learning defects and impairments in strategy choice during behavioural assays [42], whereas PSD-95 knockouts show more severe learning defects [43]. These mammalian gene specific roles in cognitive function are not found in Drosophila since the single Dlg gene is required for viability and the phenotype of loss of Drosophila NR2 is unknown [44]. Together these studies make a strong case that the vertebrate combinations of NR2 and MAGUK paralogues that form key components of synaptic signalling complexes contribute to the repertoire of cognitive behaviours. This model implies that invertebrates do not have the same signalling diversity and thus behavioural repertoire, which is consistent with comparative studies of learning [1]. Our model that evolution of behaviour is impacted by evolution of complexity of signalling complexes adds to the standard model that points to the numbers of neurons as the determinant of behavioural evolution. Importantly, the elongated NR2 together with the vertebrate specific NR2 duplication would have preceded the anatomical enlargement of brain size associated with cognitive capacities of higher animals [2].

### NR2 C-terminal dynamics

The analysis of the C-terminus structure suggests a dynamic switching function of NR2 where the domain alternates between unfolded and folded states, and that this modulates interactions with other proteins. The observed high composition of both serine and proline is a characteristic of intrinsically disordered proteins [26], which are natively unfolded and have been associated with 'hub' proteins in protein-protein interaction networks [45]. The adoption of a tertiary conformation, necessary for formation of a signalling complex, may occur following binding to intracellular adaptors, for example the PDZ domain containing proteins and CaMKII. This may be analogous to a mechanism suggested for Kv channels, where a PDZ binding domain preceded by an intrinsically disordered intracellular C-terminus facilitates structural flexibility as well as robust interaction with the scaffold protein [46,47]. Since intrinsically unfolded proteins undergo a conformational change from unfolded to folded when bound to another protein [48], the NR2 C-terminal domain should be able to form multiple conformations depending on various intracellular binding partners e.g. binding to PSD-95 induces the cytoplasmic domain to fold into a particular 3D conformation "A" and binding to CaMKII induces an alternative conformation "B". PSD-95 and CaMKII might act as cytoplasmic 'ligands' for the NMDA receptor, in an analogous manner to extracellular ligands such as Zn^+^, H^+^, and glutamate [46], and have allosteric effects on the the conformational state of NR2. Consistent with this, the interaction of PSD-95 with NR2B is known to modulate NMDA receptor channel function [49]. By binding to the NR2 C-terminal domain, these cytoplasmic ligands may in turn control the overall function of NMDA receptors (or NMDA receptor complex) as a signalling apparatus.

This 'structural switch' model implies an evolutionary selection on the unfolded domain. An alternative explanation is that there is no structural constraint and therefore nucleotides are free to be substituted rapidly by neutral evolution without any structural implications. We consider this scenario unlikely for the following reasons. Amino acid sequences for the C-terminal domain in each particular NR2 paralogues are well conserved between vertebrates, implying that since divergence of the NR2 paralogues each subunit has acquired structures that underwent purifying selection in vertebrates. Furthermore, all (~10) known phosphorylation sites and protein-protein interaction sites when mapped onto sequence alignments for each of NR2A and NR2B were found to be conserved throughout mammals. These interaction sites would require a certain extent of structural constraint, and so their conservation implies a structural framework to the NR2 C-terminal domain. Finally there is no explanation why the predicted secondary structures for NR2A and NR2B C-terminal domains have remained structurally similar since they have diverged, given that they exhibit such low similarity at the primary sequence level. The above arguments taken together suggest that a functional adaptation occurred in vertebrate evolution that accounts for NR2 C-terminal structure. Within vertebrate evolution there is evidence of further evolutionary adaptation of NR2A, which was reported to be under significant positive selection in primates when compared to rodents [50].

To test these evolutionary models it will be necessary to characterise NMDA receptor associated proteins of an invertebrate model in a comparable manner to that achieved in mice [8,35]. Detailed experimental study on the structure of the C-terminal domains using methods such as X-ray crystallography and nuclear magnetic resonance spectroscopy will be essential to test our structural predictions [47], as well as to further understand how the NMDA receptor's intracellular domain structure mediates its role in the functional organization of the NRC. More broadly, gene targeting should be employed to generate new mouse models of the NR2 C-terminus with point mutations of intracellular ligand interaction sites, paralogous swaps, and trans-species chimeras to study the molecular evolution of NMDA receptor signalling *in vivo*.

## Conclusion

NMDA receptors display a vertebrate specific elongation of the intracellular C-terminus of the NR2 subunit. This extension is unique among ionotropic glutamate receptors to the NMDA receptor NR2 subunits. Significant diversity in the NR2 C-terminus exists at the sequence level between the four NR2 paralogues in vertebrates, though each individual paralogue is highly conserved amongst non-teleost vertebrates. In contrast to the extracellular and transmembrane domains, each NR2 C-terminus is predicted to be unfolded in its native state. NR2A and NR2B C-termini are predicted to be highly similar at the level of secondary structure though they are a poorly conserved at the primary sequence level. We postulate that evolution of this vertebrate specific C-terminal domain of NR2 has resulted in an unfolded but conserved modular structure that may have contributed to the evolution and organisation of postsynaptic signalling complexes in vertebrates.

## Methods

### Sequence data & alignments

We obtained protein sequences for all genes from Ensembl Version 36. Homologues of mouse sequences were obtained using Ensembl BioMart [51]. *Schistosoma mansoni *nucleotide sequences were obtained from the Wellcome Trust Sanger Institute Pathogen Sequencing Unit's datababse [52,53]. Multiple sequence alignments we carried out using ClustalW [54]. Alignments were shaded using GeneDoc [55]. Where multiple transcripts are predicted, only a single transcript is aligned. Dotplots were generated using the Dotmatcher program in the EMBOSS package [56]. For comparing the length of C-terminal regions of multiple species, the C-terminus of each protein sequence was defined as the amino acid sequence distal of the last transmembrane residue as predicted by TMHMM [57,58].

To generate Nucleotide alignments to find the best region of similarity, the Smith-Waterman algorithm Water with an EBLOSUM62 matrix was applied [56]. Phlyogentic tree was constructed using PhyML [59].

### Protein interaction data

Protein interaction data for each NR2 subunit in mammals were obtained from the Human Protein Reference Database [7].

### Similarity searches

The nucleotide sequence of the intracellular tail of each mouse NR2 subunit were searched against non-redundant nucleotide collection, whole genome shotgun reads, and expression sequence tag databases in Genbank by BLASTn, tBLASTn, and discontiguous megablast similarity searches, with low complexity masking [60]. To search for conserved domains, the protein sequence of the intracellular tail of each mouse NR2 subunit was searched against the Pfam database using the default parameters [25].

### Structure predictions

Protein folding predictions were made using the FoldIndex graphic web server [27]. Prediction of secondary structure was made using PSIPRED Protein Structure Prediction System [28].

## Authors' contributions

Conceived and designed study: TJR, RDE, SGNG, NHK. Performed the analysis: TJR, RDE, NHK. Wrote the paper: TJR, RDE, SGNG, NHK. All authors read and approved of the final manuscript.

## Supplementary Material

Additional file 1 Smith-Waterman Alignment of Mouse NR2B C-terminal Exon Nucleotide Sequence against D. melanogaster C-terminal 2857 Nucleotide Intron. Mouse exon sequence named 'NR2B', D. melanogaster intron sequence named 'intron'. Click here for file

Additional file 2 FoldIndex Comparison of NR2 C-terminal Domain Folding Propensity. Plot of AA sequence of N-terminus (N) to C-terminus (C) of mouse NR2C and NR2D against probability of folding. Green areas are predicted to be intrinsically folded, red areas are predicted to be intrinsically unfolded. Click here for file

Additional file 3 PSIPRED Secondary Structure Predictions of Mouse NR2C & NR2D C-terminal Domains. Green barrels represent alpha helices while yellow arrows signify beta sheets. Prediction confidence represented by blue bars. Click here for file

Additional file 4 Unrooted Phylogenetic Tree of NR2 Subunits. Human (Hs), mouse (Mm), rat (Rm), zebrafish (Dr), ciona (Ci), Drosophila (Dm), C. elegans (Ce). Bootstrap values are shown at branch points. Click here for file
