# Supplementary figures and images for: Evolution of NMDA receptor cytoplasmic interaction domains: implications for organisation of synaptic signalling complexes
Source: BMC Neurosci. 2008 Jan 15;9:6. doi: 10.1186/1471-2202-9-6 (PMC2257970; doi:10.1186/1471-2202-9-6)

## Slide 1
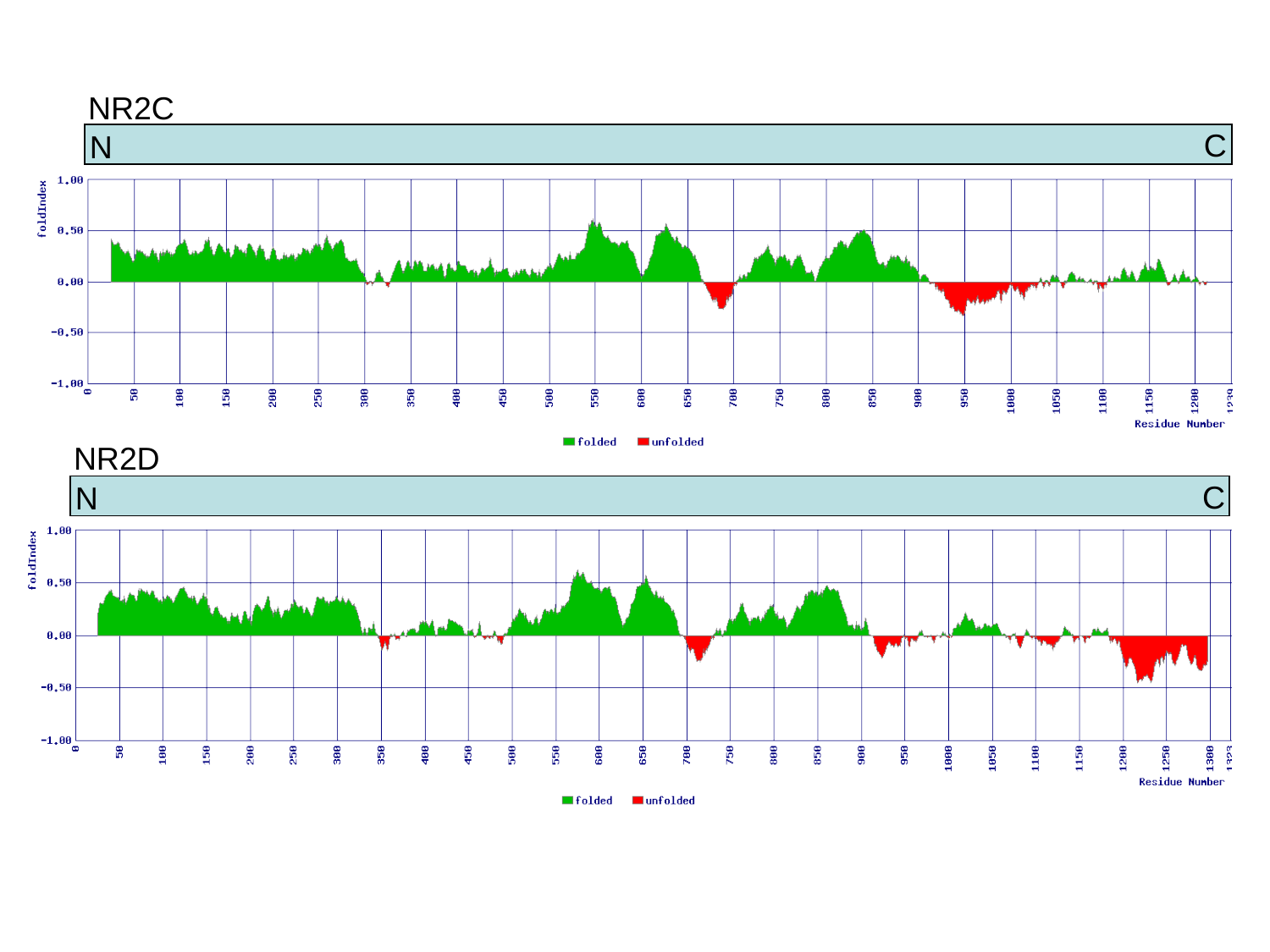

NR2C
C
N
NR2D
C
N

Supplement: Additional file 2 — FoldIndex Comparison of NR2 C-terminal Domain Folding Propensity. Plot of AA sequence of N-terminus (N) to C-terminus (C) of mouse NR2C and NR2D against probability of folding. Green areas are predicted to be intrinsically folded, red areas are predicted to be intrinsically unfolded. [file 1471-2202-9-6-S2.ppt]

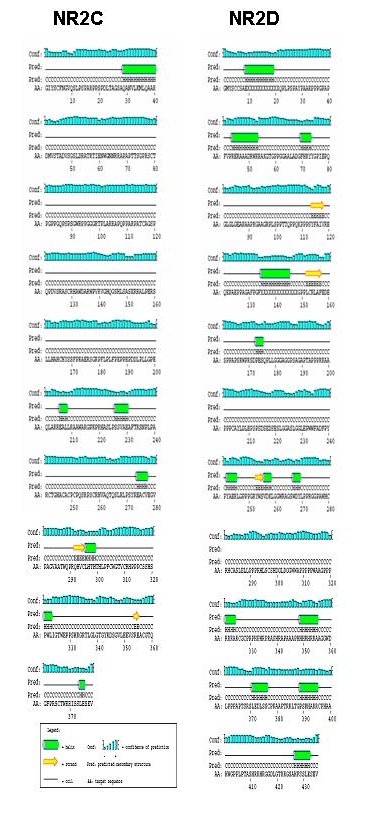

Supplement: Additional file 3 — PSIPRED Secondary Structure Predictions of Mouse NR2C & NR2D C-terminal Domains. Green barrels represent alpha helices while yellow arrows signify beta sheets. Prediction confidence represented by blue bars. [file 1471-2202-9-6-S3.JPEG]

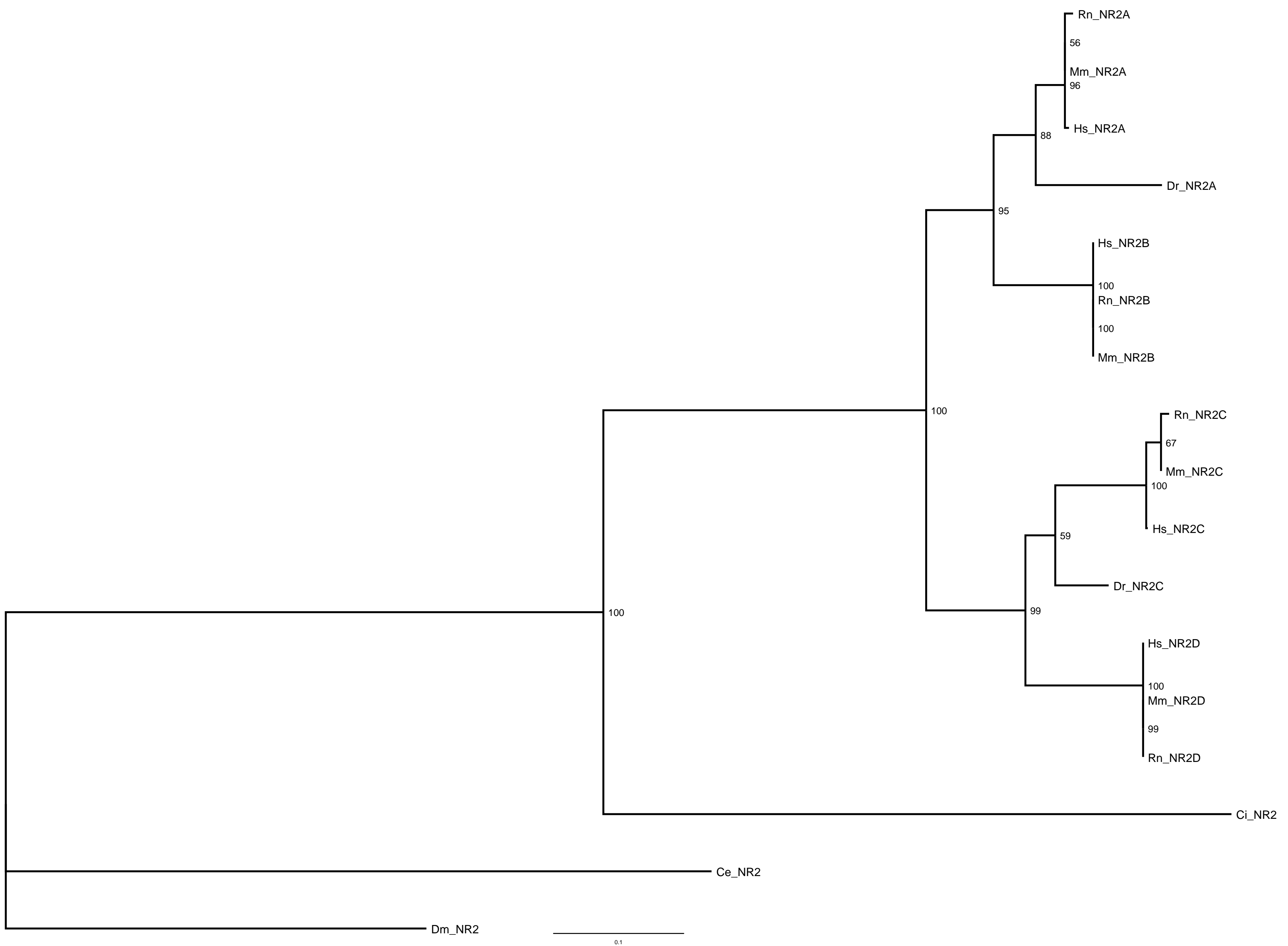

Supplement: Additional file 4 — Unrooted Phylogenetic Tree of NR2 Subunits. Human (Hs), mouse (Mm), rat (Rm), zebrafish (Dr), ciona (Ci), Drosophila (Dm), C. elegans (Ce). Bootstrap values are shown at branch points. [file 1471-2202-9-6-S4.pdf]
